# Sensibility and taste alterations after impacted lower third molar extractions. A prospective cohort study

**DOI:** 10.4317/medoral.17890

**Published:** 2012-02-09

**Authors:** Lourdes Ridaura-Ruiz, Rui Figueiredo, Eduard Valmaseda-Castellón, Leonardo Berini-Aytés, Cosme Gay-Escoda

**Affiliations:** 1 DDS. Master degree program in Oral Surgery and Implantology. University of Barcelona Dental School; 2 DDS. Associate Professor of Oral Surgery. Professor of the Master degree program in Oral Surgery and Implantology. University of Barcelona Dental School. Researcher of the IDIBELL group; 3 DDS, PhD. Professor of Oral Surgery. Professor of the Master degree program in Oral Surgery and Implantology. University of Barcelona Dental School. Researcher of the IDIBELL group; 4 MD, DDS, PhD. Professor Emeritus of Oral and Maxillofacial Surgery. Professor of the Master degree program in Oral Surgery and Implantology. University of Barcelona Dental School. Researcher of the IDIBELL group; 5 MD, DDS, PhD. Chairman of Oral and Maxillofacial Surgery. Director of the Master degree program in Oral Surgery and Implantology. University of Barcelona Dental School. Oral and maxillofacial surgeon of the Teknon Medical Center, Barcelona (Spain). Researcher of the IDIBELL group

## Abstract

Objectives: To determine the incidence, severity and duration of lingual tactile and gustatory function impairments after lower third molar removal. Study Design: Prospective cohort study with intra-subject measures of 16 patients undergoing lower third molar extractions. Sensibility and gustatory functions were evaluated in each subject preoperatively, one week and one month after the extraction, using Semmes-Weinstein monofilaments and 5 different concentrations of NaCl, respectively. Additionally, all patients filled a questionnaire to assess subjective perceptions. Results: Although patients did not perceive any sensibility impairments, a statistically significant decrease was detected when Semmes-Weinstein monofilaments. This alteration was present at one week after the surgical procedure and fully recovered one month after the extraction. There were no variations regarding the gustatory function. Conclusions: Lower third molar removal under local anesthesia may cause light lingual sensibility impairment. Most of these alterations remain undetected to patients. These lingual nerve injuries are present one week after the extraction and recover one month after surgery. The taste seems to remain unaffected after these procedures.

** Key words:**Lingual nerve, third molar, nerve injury, paresthesia, surgical extraction

## Introduction

The extraction of lower third molars (3M) is one of the most frequent surgical procedures in Dentistry. Among the more relevant risks associated with this operation are the injury of the peripheral somatosensory branches of the trigeminal nerve, mainly that of the lingual and inferior alveolar nerves (1-5).

The taste impulses initiated in the anterior area of the tongue (lingual V) are transmitted to the medulla oblongata through the gustatory fibers that are first integrated in the lingual nerve (LN), branch of the mandibular division of the trigeminal nerve. After passing through this nerve they leave to form part of the chorda tympani (CT), branch of the facial nerve. Due to the anatomical course of the LN, the gustatory fibers are in close proximity to the 3M, near the mandibular lingual cortical plate, making this area especially susceptible to surgical trauma (1,2). It is possible that some gustatory fibers arising from the tongue also reach the brain stem through the mandibular branch of the trigeminal nerve. The presence of this alternative pathway may explain the reported cases of unilateral loss of taste after sectioning the root of the trigeminal nerve.

The rate of postextraction lesions of the LN varies between 0.6 and 2% (3). In a retrospective study carried out in our department (4) based in 4,995 extractions of 3M the figure was lower than that usually published, probably due to the surgical technique that avoided the retraction of the lingual flap. This complication has also been related with anatomic factors (proximity of the lingual nerve to the lingual cortical plate) and with the surgeon’s experience (5). Another potential risk factor is the use of some local anesthetics in inferior alveolar blocks as shown in a recent paper (6). However, very few prospective studies use objective methods to assess the sensory functions of this nerve. The result is that only those lesions perceived by the patients (i.e. the most severe) are identified and included in the samples, hence underestimating the incidence of these complications. It was therefore decided to perform a study with the followings aims: to determine the incidence, severity and duration of lingual tactile and gustatory function impairments after lower third molar removal, using Semmes-Weinstein monofilaments, NaCl solutions and a neurosensory questionnaire.

## Material and Methods

A pilot prospective observational cohort study was made in 16 consecutive patients undergoing a surgical extraction of a bony impacted lower 3M in June 2007 in the Oral Surgery and Implantology department of the University of Barcelona.

The main inclusion criteria were patients without significant systemic pathologies (ASA I or II) of either gender requiring extraction of an impacted lower third molar. The exclusion criteria were the following: age over 55 years; diabetes and endocrine pa-thologies, immunosuppression, cardiovascular pathologies, hypertension; nutritional and/or neurological alterations, patients with salivary gland pathology (hyposalivation, xerostomia, etc.); patients under pharmacologic treatment or with pre or postextraction antibiotic therapy other than beta-lactamic; consumption of tobacco and/or alcohol; and having used any mouthwash one month prior and one month after the extraction.

The clinical variables collected were: age, gender, weight, soft tissue and bone coverage (none, partial or total) of the 3M (4) and mouth opening (interincisal distance assessed with a caliper) (7). The position of the third molar was determined on panoramic radiographs following the Pell and Gregory and Winter classifications.

The patients were informed of the objectives of the study and gave their informed consent. The study was approved by the Institutional Review Board of the Dental Clinic of the University of Barcelona. The authors have read the Helsinki Declaration and have followed the guidelines in this investigation.

Gustatory perception test

**Figure 1:**
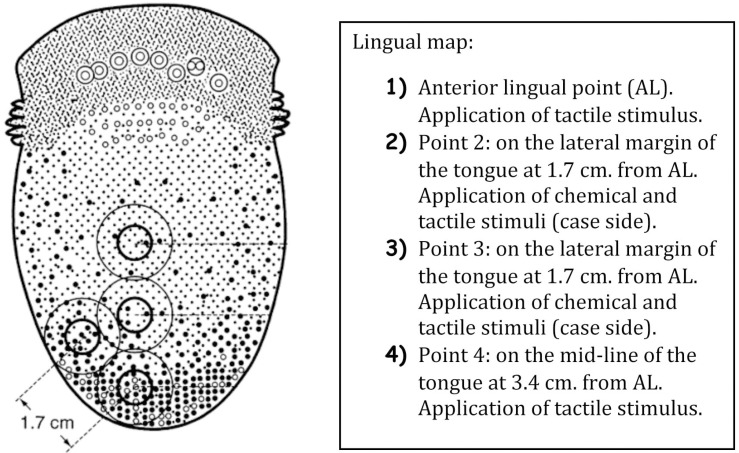
Lingual map indicating the location of the applied taste and sensibility stimuli.

This function was evaluated using 5 solutions with different concentrations of NaCl (0.01 mol/L; 0.51 mol/L; 1.01 mol/L; 1.51 mol/L; 2.01 mol/L) presented in samples of 5 ml. The anterolateral border of the tongue was explored bilaterally beginning with the operated side (case side) (Fig. 1). The first solution to be used was the one with the smallest concentration. A 5 mm diameter filter paper disk was impregnated and maintained in the area for a maximum of five seconds. In the event of a lack of response, the patient was instructed to rinse the mouth with water and the test was repeated after 30 seconds with a solution of progressively higher concentration, until the taste was recognized and differentiated. The non-operated site was used as a control.

Objective sensibility tests

Lingual sensibility was evaluated in each patient by means of Semmes-Weinstein monofilaments (SW test) (Touch-TestTM; North Coast Medical, Morgan Hill, United States). This function was studied preoperatively, and at one week and one month after the extraction. The filaments, starting with the one of smallest diameter, were applied bilaterally and perpendicular to the tongue until bending, thus delivering the target force ( Table 1). The patients were instructed to close their eyes and to raise their hand when pressure was detected. In the event of a negative response, the test was repeated after 30 seconds with a larger diameter monofilament, until the stimulus was recognized. The evolution of sensibility was obtained comparing the thickness of the monofilament detected by the patient at the different evaluations. In addition, sensitivity to pain and directional discrimination were measured using a dental probe. The lingual map used for the examinations is shown in (Fig. 1). This examination was repeated bilaterally (the non-operated site was used as control).

**Table 1:**
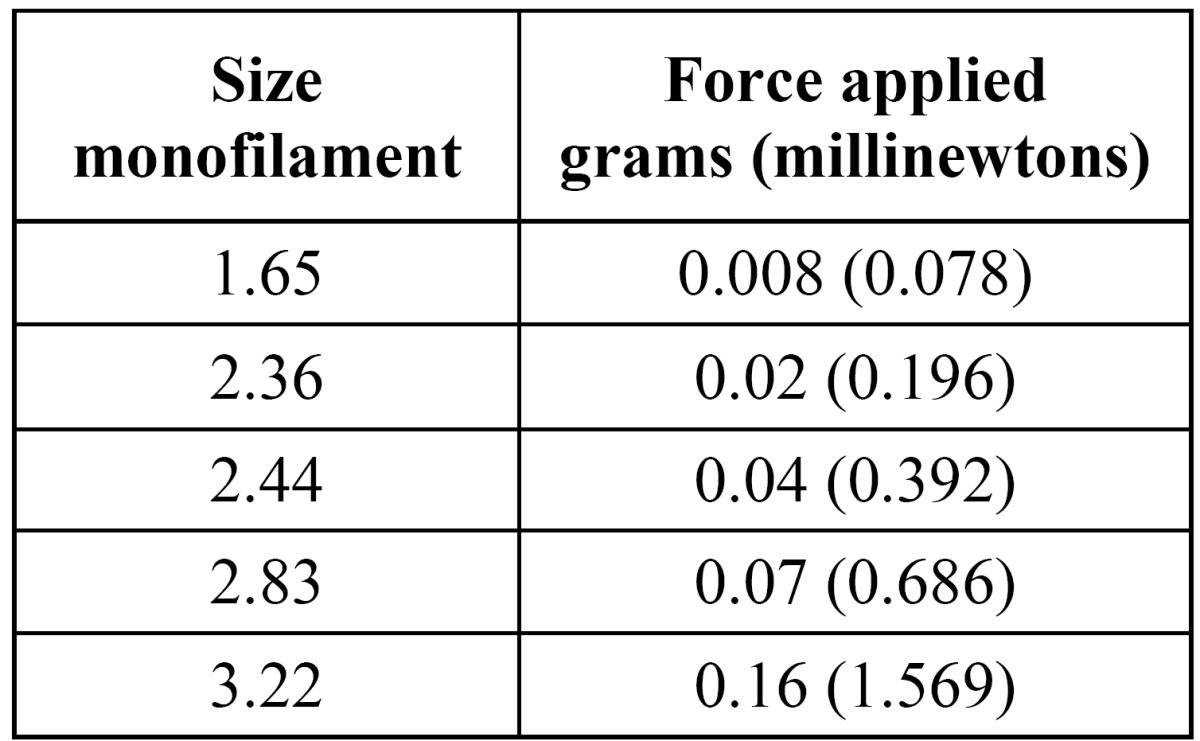
Force (measured in grams or in millinewtons) applied by the Semmes-Weinstein monofilaments according to its diameter.

Neurosensory questionnaire

The patient subjectively evaluated the sensibility of the tongue, and/or the changes in taste by means of a questionnaire composed by 7 questions (2 related with sensitivity and 5 with taste) ( Table 2). The subjects filled this questionnaire at one week and one month after the surgical procedure.

**Table 2:**
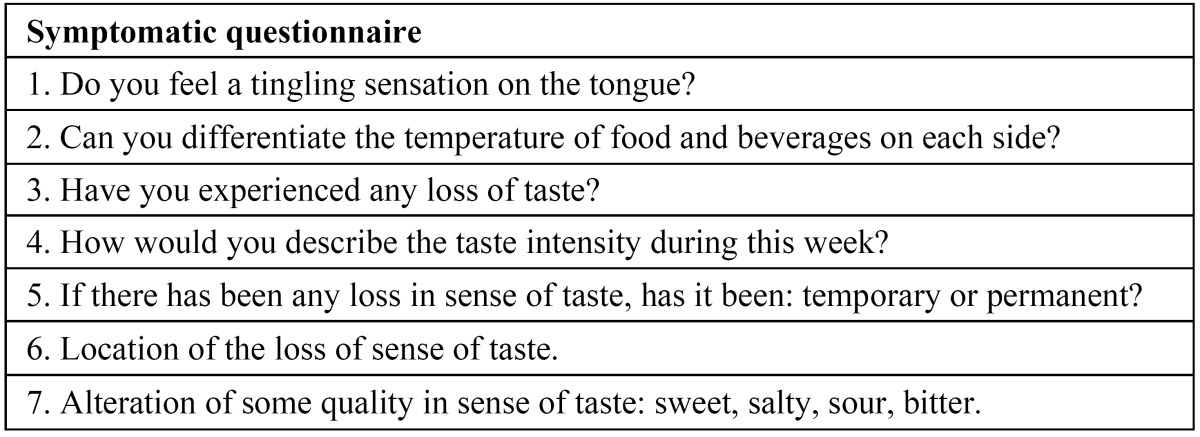
Neurosensory questionnaire.

Surgical procedure

All patients had one lower 3M extracted in each operation, under local anesthesia with articaine in a 4% solution with epinephrine 1:100.000 (Artinibsa; Inibsa, Lliça de Vall, Spain). The surgical field and all the surgical material were sterile. One single surgeon raised a full-thickness flap, which was protected by a Minnesota retractor. Lingual flap retraction was not performed since the surgeon considered it to be unnecessary. Sterile low-speed (20.000 rpm) handpieces and sterile saline solution were employed for bone removal and tooth sectioning when necessary. To close the wound, 3-0 silk sutures (Silkam, Braun; Tuttlingen, Germany) were used. After 7 days, a surgeon removed the sutures.

After the operation, an antibiotic (amoxicillin 750 mg every 8 hours for 7 days [Clamoxyl 750; GlaxoSmithKline, Madrid, Spain]), a nonsteroidal anti-inflammatory drug (sodium diclofenac 50 mg every 8 hours [Diclofenaco Llorens 50 mg; Llorens; Barcelona, Spain] for 4-5 days) and an analgesic (metamizol 575 mg every 6 hours for 2-3 days [Nolotil; Boehringer Ingelheim; Sant Cugat del Vallès, Spain]) were prescribed. The patient rinsed with physiological serum twice a day for 15 days. Postoperative instructions and prescribed drugs were explained and printed on a paper that was given to the patient.

Statistical analysis

The data were processed with the Statistical Package for the Social Sciences version 12.0 for Windows (SPSS; SPSS Inc.; Chicago, Ill, U.S.). An analysis of variance (ANOVA) for repeated measures was carried out, using Greenhouse-Geisser correction when sphericity did not hold. Differences between subgroups at 1 week and 1 month after surgery were checked additionally with t-tests for paired samples.

## Results

Sixteen patients, 5 women (31.25%) and 11 men (68.75%) were included. The mean age was 26.9 years (standard deviation (SD) of 11.2 years) and the mean weight was 64.9 kg (SD = 11.8 kg). Most patients (n=11) had the left 3M removed. The positions of the extracted teeth according to the Pell & Gregory and Winter classifications are displayed in ( Table 3). All extracted third molars required bone removal and tooth sectioning.

**Table 3:**
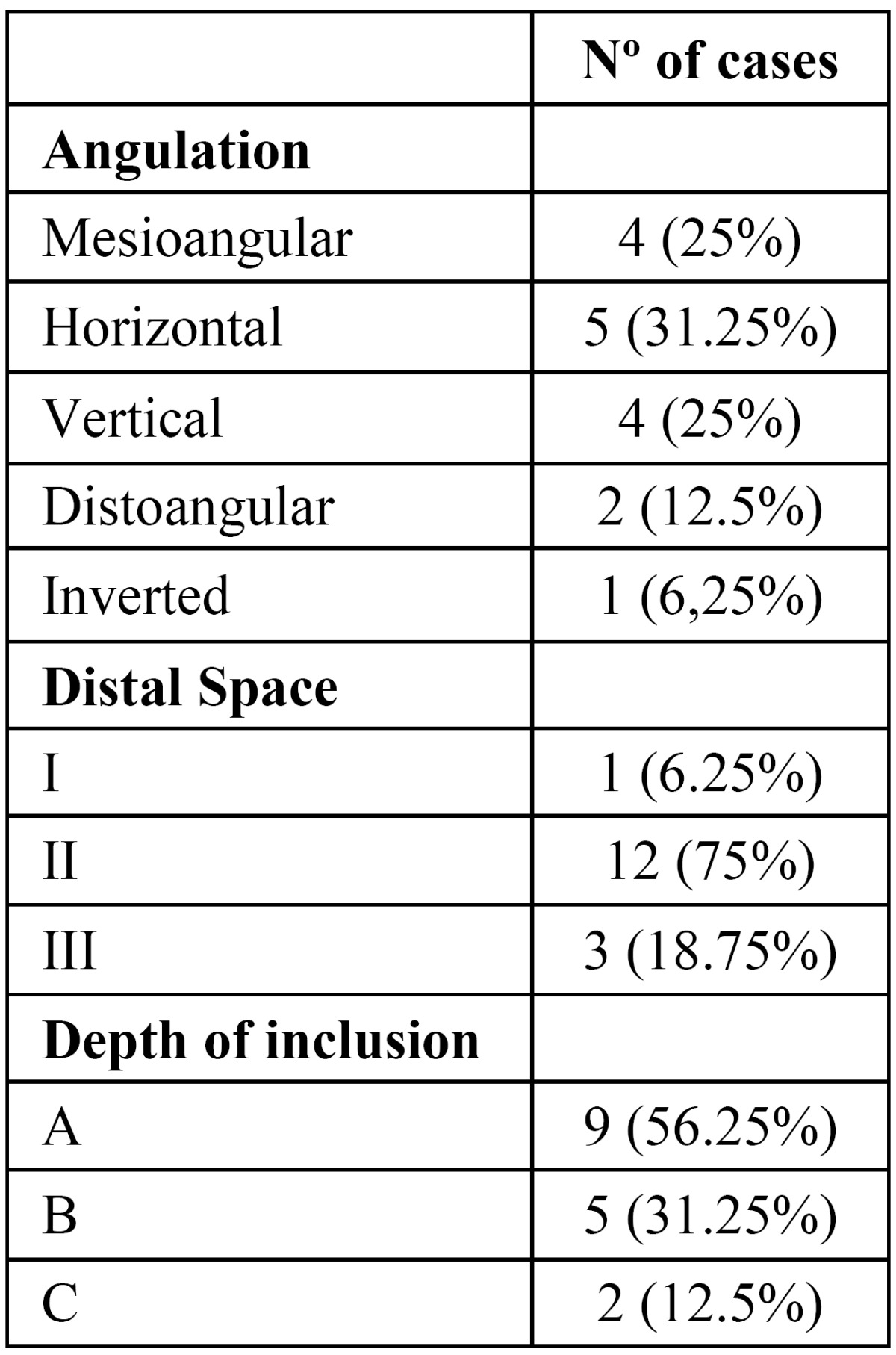
Position of the lower third molar according to Pell & Gregory and Winter classifications.

Patients showed a significant change with time regarding mouth opening (ANOVA for repeated measures: F=36.184; df=1.277; p=2.73•10-6). One week after the extraction, the mean reduction was of 8.63 mm (SD=1.313) that was statistically significant (p=8.90•10-6). At the final observation, the difference with the baseline was non significant (p=0.165).

Gustatory perception test

No significant differences were found between the values obtained on both sides (ANOVA for repeated measures: F=3.462; df=1; p=0.083). The measurements over time for each individual side were similar (ANOVA for repeated measures: F=0.550; df=1.331; p=0.516), observing only a slight increase in the recognition threshold, 7 days after the surgical procedure. The evolution of the gustatory function was similar for both sides (ANOVA for repeated measures: F=0.484; df=1.208; p=0.530) ( Table 4).

**Table 4:**
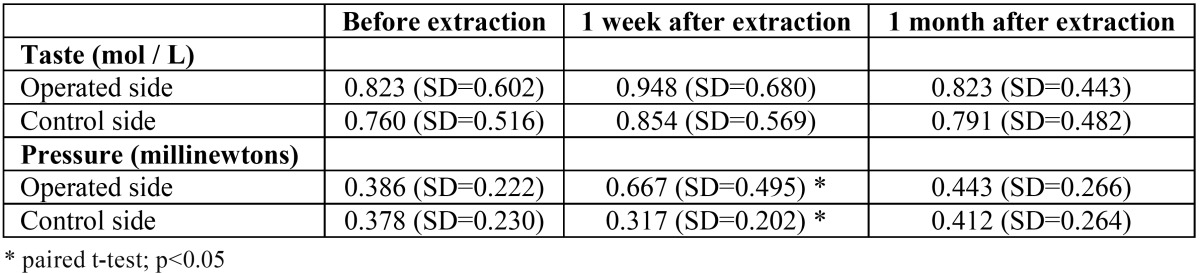
Means and standard deviations of the minimal detected concentration of NaOCl (paired t-tests: p>0.05) and of the force applied with the monofilaments on the case and control sides over time. At 1 week post-extraction the detection threshold of the operated side was 0.349 millinewtons higher (SD=0.096 millinewtons) than that of the control side (paired samples t-test: t=3.623; gl=15; p=0.003).

Objective sensibility test

The mean values for the detected monofilaments (ANOVA for repeated measures: F=16.721; df=1; p=0.001) were found to be significantly higher on the experimental side. There was a statistically significant relationship (ANOVA for repeated measures: F=10.002; df=1.126; p=0.005) between the time intervals of monofilament recognition and side: 7 days after extraction, on the operated side, a wider diameter monofilament was necessary to reach the detection threshold. One month after lower third molar removal, the values were similar ( Table 4). No deficits were detected in directional discrimination or sensitivity to pain.

Neurosensory questionnaire

None of the patient referred subjective alterations in either sensibility or gustatory functions. The patients did not perceive the variations registered with the monofilaments.

## Discussion

Several nerves responsible for the transmission of general sensory stimulus may be damaged during dentoalveolar surgery procedures. Although these complications are infrequent after the extraction of lower 3M, alterations have been widely described (3-5). A nerve injury may be caused by injection of local anesthetic as a result of mechanical (by direct contact with the needle) or chemical (due to the neurotoxic effects of the anesthetic compounds) action (1). However, in a retrospective analysis of paresthesias diagnosed after injection of local anesthetic, it was observed that the estimated incidence was extremely low (1:785,000) (8). A more recent study (6), based on the U.S. Food and Drugs Administration Adverse Event Reporting System, confirmed this low figure, with only 248 nerve injuries being identified in an 11 years period. These authors state that in 89% of cases the lingual nerve was involved (6). A possible explanation to this outcome is that this nerve is more exposed to needle contact and presents a smaller number of fasciculi when compared to the inferior alveolar nerve (3). Several reports, have claimed that articaine and prilocaine might have a neurotoxic effect due to its high concentration, thus increasing the incidence of sensory impairments after inferior alveolar nerve blocks (6,8). In our study, a 4% articaine solution was used in all patients and this has to be taken into consideration when analyzing the outcomes. In our opinion, it is unlikely that the neurosensory deficits observed in our sample were caused by the local anesthetic, especially because of the published incidence. Nevertheless, a randomized clinical trial comparing the effect of local anesthetics (lidocaine and articaine) on the incidence of nerve injuries after inferior alveolar nerve blocks would be of great interest. From a methodological point of view, this might be extremely difficult to perform since this is an extremely rare event. However, the use of objective methods to assess the sensibility might reduce the need for an extremely large sample, as shown by our results.

Shafer et al. (1) in 1999, published a study to evaluate the gustatory function before and after the extraction of third molars. These authors studied the taste capacity using solutions of NaCl, saccharose, citric acid and quinine hydrochloride and found that the most frequent alteration was a deficit in taste intensity. They indicated that this reduction, which persisted for at least six months, was probably related to nerve compression or laceration, secondary to surgical trauma and/or edema, since the depth of impaction of the molar was significantly related to taste deficits. Akal et al. (2) published a study with a similar design but with opposing results. Likewise, in our study no significant changes in taste were observed and only a slight increase in the detection threshold was found 7 days after surgery. The fact that only NaCl solutions (and with small concentrations increases) were used, might partially explain the lack of significant differences between the groups. Another important factor is the small sample size of our study. Nevertheless, if the present results were used to make a power analysis (α=0.05; β=0.2; effect size f=0.0783), over 800 patients would be needed to detect a significant difference, which clearly makes the development of such study extremely difficult.

To evaluate sensibility, Semmes-Weinstein monofilaments were used. These instruments are individually calibrated nylon mono-filaments of different thicknesses that transmit a specific force. The number of each monofilament represents the tenth logarithm of the force in milligrams or millinewtons ( Table 1) necessary to curve it (force log10 [0.l mg]) when applied to a surface (9). It is an exact and noninvasive method, employed in neurosensory evaluation of corporal sensitivity, and that can also be used to assess the evolution of nerve lesions (9-11). The sensibility level of the area innervated by the trigeminal nerve is considered normal when the patient is able to detect monofilaments of diameters 1.65 and 2.36 (10,12,13). Nevertheless, these values are influenced by several factors like age, gender and race, which complicate the interpretation of the results (14,15). In the present study, each patient acts as its own control, thus reducing the effect of these variables and increasing the statistical power.

The alterations in taste and/or touch are also related to the specific areas of the LN that have been damaged. Watanabe et al. (16) demonstrated a higher frequency in the lesion of the lateral fibers with respect to the medial ones. The fibers forming part of the CT maintain a more superficial and posterolateral location in the LN. Hence, according to this observation it would be more likely to observe dysfunction of gustatory perceptions rather than sensibility alterations.

The relation between the objective neurosensory deficits and those referred subjectively by the patients appears to be questionable. Some studies have found that subjects seem to report higher sensory deficits, than those recorded with objective methods (11,17). On the other hand, Coghlan and Irvine (18) indicate that patients refer less neurosensory alterations than in the objective tests. The fact that the subjects included in these papers (11,17,18) were submitted to orthognatic surgery might make comparisons inaccurate, since the type of injuries and also the affected nerve are different. In our study, objective sensibility changes remained undetected to the patients. This indicates that lower third molar removal might produce very slight injuries resulting in clinically insignificant paresthesias that fully recover in a brief period of time. However, this fact should be taken into consideration for future investigations, since clinically undetectable lesions seem to exist, and therefore the incidence may vary between studies in function of the methods used to evaluate sensibility.

## Conclusion

Lower third molar removal under local anesthesia may cause light lingual sensibility impairment that generally can only be assessed by means of objective tests. Most of these alterations remain undetected to patients. These lingual nerve injuries are present one week after the extraction and recover one month after surgery. The taste seems to remain unaffected after these procedures.
